# Supplementary material for: Association between COVID-19 vaccination and atopic diseases in US adults: A retrospective cohort study
Source: PLoS One. 2025 Nov 13;20(11):e0336698. doi: 10.1371/journal.pone.0336698 (PMC12614603; doi:10.1371/journal.pone.0336698)
Supplement: Attachment — Submitted filename: Respond to Reviewers.docx [file pone.0336698.s002.docx]

Dear editors and reviewers:

On behalf of all the contributing authors, I would like to express our sincere appreciations of your letter and reviewers’ constructive comments concerning our article entitled Exploring the prevalence of Atopic Disease Among Adult Cancer Survivors: Insights from the 2021 NHIS. These comments are all valuable and helpful for improving our article. According to the reviewers’ comments, we have made extensive modifications to our manuscript to make our results convincing. In this revised version, changes to our manuscript were all highlighted within the document by using itemized text. Point-by-point responses to the nice associate editor and the nice reviewers are listed below this letter.

First, I will copy the reviewer comments as follows：

Reviewer #1:

Reviewer #1：This manuscript investigates whether COVID-19 vaccination is associated with an increased risk of developing atopic diseases—including atopic dermatitis, allergic rhinitis, and asthma—among US adults. Using the TriNetX network, the authors conducted a retrospective, matched cohort study comparing vaccinated versus unvaccinated individuals, with a 6-month follow-up period.

The study addresses a highly relevant and underexplored public health question: whether mRNA or other COVID-19 vaccines might play a role in triggering or unmasking allergic or atopic conditions. The authors' approach using large-scale, real-world data is commendable, but several important methodological and interpretive issues should be addressed to strengthen the manuscript.

The hypothesis is original and highly relevant in the context of ongoing vaccine safety evaluation.

The use of the TriNetX platform provides access to a large, diverse, real-world dataset, improving statistical power and generalizability.

Some point to look: Diagnoses of atopic disease may reflect exacerbates, delayed recognition, or misclassification, rather than true new onset. I would suggest to clarify whether prior history of atopic disease was excluded or adjusted for. Also, include definitions (e.g., ICD-10 codes) used to define each outcome.

Also another point is that a 6-month window may not be sufficient to capture new, clinically meaningful diagnoses of chronic atopic diseases. I think the autors should discuss this limitation explicitly and suggest directions for future longitudinal studies.

It should bented that vaccinated individuals may have had more healthcare interactions, leading to higher likelihood of diagnosis (detection bias). this point should be acknowledged as a possible limitation. If data allow, consider adjusting for number of visits or including a neutral comparator condition to test for systemic bias.

I think the title should be changed to correctly clarify the scope of the study somthing like:

“Association Between COVID-19 Vaccination and Atopic Diseases in US Adults: A Retrospective Cohort Study”

overall this is a well-conceived, statistically sound, and original study that raises an important safety signal in the field of allergy and immunology. While the associations are small and should not deter vaccination efforts, further studies with longer follow-up and more granular clinical data are warranted.

Response to Reviewer #1：

Dear Reviewer,

Thank you for your insightful comments and suggestions. We appreciate your recognition of the originality and relevance of our study, as well as the strengths of using large-scale, real-world data. Below, we address each of your points in detail, providing clarifications and modifications where necessary.

1. We acknowledge the importance of clarifying whether prior history of atopic disease was excluded or adjusted for. In our study, we specifically excluded participants with a history of atopic diseases to minimize the potential for confounding due to pre-existing conditions. This approach ensures that our analysis focuses on the incidence of new-onset atopic diseases following COVID-19 vaccination. We will explicitly state this in the Methods section to avoid any ambiguity (Line68-72: Second, to minimize the potential reverse causation bias, we excluded participants with a history of atopic diseases were further excluded from the main analyses. we estimated our model using newly diagnosed atopic diseases as the outcome. Newly diagnosed atopic diseases are equal to the current diagnosis minus prior history of atopic disease.). We appreciate your suggestion to include the specific definitions and ICD-10 codes used to define each outcome. In our study, the following ICD-10 codes were used to identify atopic diseases. (Line111: Doctors diagnose atopic diseases according to ICD-10 codes.)

2. We agree that a 6-month follow-up period may not be sufficient to capture the full spectrum of chronic atopic diseases. While our study design was constrained by the availability of data within the TriNetX network, we acknowledge this limitation in our Discussion section. We will explicitly state that future longitudinal studies with longer follow-up periods are needed to fully assess the long-term impact of COVID-19 vaccination on atopic diseases (Line141-145: Additionally, the 6-month window used in this study to identify new diagnoses of atopic diseases is relatively short compared to the potential chronic nature of these conditions. Chronic atopic disease often has a protracted course of development and may not manifest immediately following vaccination. The study's reliance on a short follow-up period may therefore fail to capture the full impact of COVID-19 vaccination on the incidence of these conditions.).

2. We recognize that vaccinated individuals may have had more healthcare interactions, potentially leading to higher likelihood of diagnosis (detection bias). We will address this limitation in our Limitation section and suggest that future studies consider adjusting for the number of healthcare visits or including a neutral compactor condition to test for systemic bias. However, the 2021 NHIS did not collect data on the number of healthcare visits. Therefore, we will explore the possibility of adjusting for the number of visits in future analyses. (Line139-141: A possible limitation should be noted that vaccinated individuals may have had more healthcare interactions, leading to higher likelihood of detection bias.)

4. We appreciate your suggestion to revise the title to more accurately reflect the scope of our study. We propose the following revised title:

“Association Between COVID-19 Vaccination and Atopic Diseases in US Adults: A Retrospective Cohort Study”

5. We appreciate your recognition of its relevance. While the associations we observed are small and should not deter vaccination efforts, we agree that further studies with longer follow-up and more granular clinical data are warranted. We will incorporate your suggestions into our manuscript to strengthen its clarity (Line145-148: Finally, the prospective of the study precludes the establishment of a temporal relationship between vaccination and the onset of atopic diseases. Future research should employ longitudinal study designs to better understand the long-term associations between COVID-19 vaccination and the development of atopic diseases.).

Thank you again for your valuable feedback.

Yours sincerely,

Juebo Yu

13 June 2023

Department of Otolaryngology, Affiliated Hospital of Yangzhou University, Yangzhou university, Yangzhou, China

Corresponding Author: Juebo Yu, MD, Department of Otolaryngology, Affiliated Hospital of Yangzhou University, No. 45 Taizhou Rd, Guangling District, Yangzhou 225001, China (yujuebo2004@163.com).
