# Supplementary material for: Association between COVID-19 vaccination and atopic diseases in US adults: A retrospective cohort study
Source: PLoS One. 2025 Nov 13;20(11):e0336698. doi: 10.1371/journal.pone.0336698 (PMC12614603; doi:10.1371/journal.pone.0336698)
Supplement: Attachment — Submitted filename: Respond to Reviewers 2.docx [file pone.0336698.s003.docx]

Dear editors and reviewers:

On behalf of all the contributing authors, I would like to express our sincere appreciations of your letter and reviewers’ constructive comments concerning our article entitled **Association between COVID-19 vaccination and atopic diseases in US adults: a retrospective cohort study**

. These comments are all valuable and helpful for improving our article. According to the reviewers’ comments, we have made extensive modifications to our manuscript to make our results convincing. In this revised version, changes to our manuscript were all highlighted within the document by using itemized text. Point-by-point responses to the nice associate editor and the nice reviewers are listed below this letter.

First, I will copy the reviewer comments as follows：

Reviewer #1:

Reviewer #1: The study, "Association between COVID-19 vaccination and atopic diseases in US adults: a retrospective cohort study", investigates whether COVID-19 vaccination is associated with the onset of atopic diseases in adults using a large, multi-institutional electronic health record database (TriNetX). By analyzing over 1.9 million vaccinated individuals and a propensity score–matched unvaccinated cohort, you assessed the incidence of atopic dermatitis, allergic rhinitis, asthma, and food allergies over a six-month follow-up. The results indicate no increased risk of developing atopic diseases post-vaccination; in fact, certain conditions such as allergic rhinitis and asthma were less common in the vaccinated group. Subgroup analyses by age, sex, and vaccine type confirmed the consistency of these findings. The work addresses an important public concern about vaccine safety and provides robust evidence that COVID-19 vaccination is not linked to higher rates of atopic disease. The results are reassuring for clinicians, patients, and policymakers and may help reduce vaccine hesitancy. In my view, the corrected manuscript addressed all the reviewers comments.

Reviewer #2: This study represents a valuable effort to investigate the association between COVID-19 vaccination and allergic diseases. I believe that addressing the following points will further enhance the quality of the manuscript.

Page 2 line 56-57
Based on the authors’ statistical definition, skin allergy appears to show a statistically significant increased risk. Therefore, the authors should revise the wording throughout the manuscript, including the main text, to ensure consistency with the statistical findings.

Page3 line 112
While the authors state that diagnoses were made according to ICD-10 codes, it remains unclear which specific codes were used to define the conditions analyzed in this study. It would be helpful and more transparent if the authors could provide a supplementary table listing the exact ICD-10 codes used for each disease definition.

Page5 line 169-172
The authors state that individuals with a history of atopic disease were excluded prior to the analysis; however, the definition of "atopic disease" used for this exclusion is not clearly provided. The authors should clearly define the specific conditions considered as atopic disease.
Additionally, it would enhance the clarity of the study if the authors could report the total number of individuals initially identified, the number excluded due to prior atopic disease, and a breakdown of the excluded cases. A flow diagram illustrating this process would be highly recommended.
Furthermore, it would be beneficial for the authors to comment on whether the proportion of participants excluded due to atopic disease was appropriate, and how this may have impacted the representativeness of the final study population.

Page5 lines 207-208　“These results …… increases.”
This sentence does not present a result and therefore should be deleted.

Figure1
It appears that not all of the factors examined in the subgroup analyses are clearly described. It is unclear which specific analysis results were used to generate the corresponding figure.
Additionally, it is difficult to determine whether the optimal predictive model presented in Table 5 has been thoroughly evaluated or considered by the authors. The authors should provide clear descriptions of the figure.

### **Response to Reviewer #1**

Dear Reviewer #1,

Thank you for your positive feedback and recognition of the importance of our study. We are pleased that you found our work to be of high quality and that it addresses a significant public concern regarding vaccine safety. We appreciate your acknowledgment that the corrected manuscript has adequately addressed all the reviewers' comments. We believe that our findings will indeed help reduce vaccine hesitancy and provide reassurance to clinicians, patients, and policymakers.

### **Response to Reviewer #2**

Dear Reviewer #2,

Thank you for your valuable comments and suggestions. We have carefully considered each of your points and have made the necessary revisions to enhance the quality of our manuscript. Below are our detailed responses to each of your comments:

#### **Page 2 line 56-57**

****Reviewer Comment:**** Based on the authors’ statistical definition, skin allergy appears to show a statistically significant increased risk. Therefore, the authors should revise the wording throughout the manuscript, including the main text, to ensure consistency with the statistical findings.

****Response:**** We apologize for the inconsistency in our wording. We have revised the manuscript to ensure that all references to the statistical findings regarding skin allergy are consistent with the results of our analysis. Specifically, we have updated the text to reflect that the adjusted odds ratio for skin allergy was 1.03 (95% CI, 0.86-1.28; P=0.135).

#### **Page 3 line 112**

****Reviewer Comment:**** While the authors state that diagnoses were made according to ICD-10 codes, it remains unclear which specific codes were used to define the conditions analyzed in this study. It would be helpful and more transparent if the authors could provide a supplementary table listing the exact ICD-10 codes used for each disease definition.

****Response:****We agree that providing the specific ICD-10 codes used for each disease definition will enhance the transparency of our study.

Doctors diagnose atopic diseases according to ICD-10 codes[L20(all sub-codes0), J30 (all sub-codes),J45 (all sub-codes),and T78.0]

#### **Page 5 line 169-172**

****Reviewer Comment:**** The authors state that individuals with a history of atopic disease were excluded prior to the analysis; however, the definition of "atopic disease" used for this exclusion is not clearly provided. The authors should clearly define the specific conditions considered as atopic disease. Additionally, it would enhance the clarity of the study if the authors could report the total number of individuals initially identified, the number excluded due to prior atopic disease, and a breakdown of the excluded cases. A flow diagram illustrating this process would be highly recommended. Furthermore, it would be beneficial for the authors to comment on whether the proportion of participants excluded due to atopic disease was appropriate and how this may have impacted the representativeness of the final study population.

****Response:**** We have clarified the definition of "atopic disease" used for exclusion in the manuscript. Specifically, we defined atopic diseases as including hay fever, asthma, skin allergy, and food allergy. We have also added a detailed description of the exclusion process, including the total number of individuals initially identified, the number excluded due to prior atopic disease, and a breakdown of the excluded cases. A flow diagram (Figure 1) illustrating this process has been included in the study. Additionally, we have commented on the appropriateness of the proportion of participants excluded and its potential impact on the representativeness of the final study population.

The results showed that The multivariable-adjusted OR for the risk of still asthma(n=2,471) was 1.12 (95% CI,1.03-1.27;P=0.011), for the risk of newly diagnosed skin allergy (n = 698; 2,496 of 3,230 total skin-allergy cases were excluded because of pre-existing disease)was 0.98 (95% CI,0.84-1.12;P=0.799) , for the risk of newly diagnosed hay fever (7,728 of 14,491 total hay-fever cases were excluded because of pre-existing disease) was 1.17 (95% CI,1.10-1.24;P< 0.001), and for the risk of newly diagnosed food allergy(n=1,084；1,838 of 2,922 total food-allergy cases were excluded because of pre-existing disease) was 1.01 (95% CI,0.89-1.15;p=0.823).

#### **Page 5 lines 207-208**

****Reviewer Comment:**** These results …… increases. This sentence does not present a result and therefore should be deleted.

****Response:**** We have removed the sentence as suggested to ensure that only relevant and accurate results are presented in the manuscript.

#### **Figure 1**

****Reviewer Comment:**** It appears that not all of the factors examined in the subgroup analyses are clearly described. It is unclear which specific analysis results were used to generate the corresponding figure. Additionally, it is difficult to determine whether the optimal predictive model presented in Table 5 has been thoroughly evaluated or considered by the authors. The authors should provide clear descriptions of the figure.

****Response:**** Thank you for your suggestion. Because the factors contributing to atopic disease are multifaceted, the figure derived from the present study lacks sufficient scientific validity; therefore, we have removed it.

Thank you again for your valuable feedback. We believe that these revisions have significantly improved the quality and clarity of our manuscript.

Yours sincerely,

Juebo Yu

20 /8/ 2023

Department of Otolaryngology, Affiliated Hospital of Yangzhou University, Yangzhou university, Yangzhou, China

Corresponding Author: Juebo Yu, MD, Department of Otolaryngology, Affiliated Hospital of Yangzhou University, No. 45 Taizhou Rd, Guangling District, Yangzhou 225001, China (yujuebo2004@163.com).
