# Supplementary material for: Association between COVID-19 vaccination and atopic diseases in US adults: A retrospective cohort study
Source: PLoS One. 2025 Nov 13;20(11):e0336698. doi: 10.1371/journal.pone.0336698 (PMC12614603; doi:10.1371/journal.pone.0336698)
Supplement: Attachment — Submitted filename: Respond to Reviewers 3.docx [file pone.0336698.s004.docx]

Dear editors and reviewers:

On behalf of all the contributing authors, I would like to express our sincere appreciations of your letter and reviewers’ constructive comments concerning our article entitled **Association between COVID-19 vaccination and atopic diseases in US adults: a retrospective cohort study**

. These comments are all valuable and helpful for improving our article. According to the reviewers’ comments, we have made extensive modifications to our manuscript to make our results convincing. In this revised version, changes to our manuscript were all highlighted within the document by using itemized text. Point-by-point responses to the nice associate editor and the nice reviewers are listed below this letter.

First, I will copy the reviewer comments as follows：

**Journal Requirements:**

1. If the reviewer comments include a recommendation to cite specific previously published works, please review and evaluate these publications to determine whether they are relevant and should be cited. There is no requirement to cite these works unless the editor has indicated otherwise. 

2. Please review your reference list to ensure that it is complete and correct. If you have cited papers that have been retracted, please include the rationale for doing so in the manuscript text, or remove these references and replace them with relevant current references. Any changes to the reference list should be mentioned in the rebuttal letter that accompanies your revised manuscript. If you need to cite a retracted article, indicate the article’s retracted status in the References list and also include a citation and full reference for the retraction notice.

**Additional Editor Comments:**

Dear Authors,

Thank you for your thorough revisions and detailed responses. The reviewers and I agree that most of the previous concerns have been adequately addressed.

However, we take Reviewer #2’s comment seriously. I have also noted that the corresponding data in the related table (as cited in the Abstract) were modified accordingly. Although this change does not appear to affect your results or conclusions, I would still appreciate a brief explanation or clarification regarding this modification in your response letter.

**Reviewer #1:** Thank you for the clarifications and answers according to all the reviewers comments. I think the article on its present form is very good anfd I recomend to accept it for publishment.

**Reviewer #2:** The authors have adequately addressed the comments. However, one point of concern remains: in lines 56-57, the numerical results have been altered without a clear explanation for this modification. If this change is appropriate and accurate, I have no additional comments.

**Response to Reviewer**  **Journal Requirements & Editor’s Statement**

**Below is our point-by-point reply to the latest editorial and journal requirements.**

**1. Reviewer-suggested citations**

**We reviewed every paper that the reviewers recommended. None were mandated by the editor; after evaluating their relevance we decided that adding them was unnecessary because our literature coverage is already complete and fully supports the statements made.**

**2. Reference-list integrity**

**The entire reference list was re-checked for completeness, accuracy, and retraction status. No retracted articles are cited; therefore no explanatory footnote or replacement was required. No references were added, removed, or re-ordered in this revision.**

**3. Numerical discrepancy in the Abstract (Editor’s specific query)**

**This change occurred because, during the first revision, we re-ran the analysis and obtained an adjusted OR of 1.03（95%CI, 0.86-1.28; P=0.135）for skin allergy. Unfortunately, we failed to explain this update promptly, and I sincerely apologize for omitting that clarification in our previous response letter. This typographical correction does not alter any conclusions.**

**Response to Reviewer** Reviewer #1 and Reviewer #2,

Thank you both for your final comments and for your recommendation to accept the manuscript.

Reviewer #1 – we are grateful for your positive assessment.

Reviewer #2 – Thank you for pointing out the remaining discrepancy in lines 56–57.
This change occurred because, during the first revision, we re-ran the analysis and obtained an adjusted OR of 1.03（95%CI, 0.86-1.28; P=0.135）for skin allergy. Unfortunately, we failed to explain this update promptly, and I sincerely apologize for omitting that clarification in our previous response letter. The values in the Abstract and Table 3 are now identical; the minimal difference does not affect our conclusions. We have double-checked every other number in the Abstract against the tables to ensure that no similar oversights remain.

Thank you again for your valuable feedback. We believe that these revisions have significantly improved the quality and clarity of our manuscript.

Yours sincerely,

Juebo Yu

20 /10/ 2025

Department of Otolaryngology, Affiliated Hospital of Yangzhou University, Yangzhou university, Yangzhou, China

Corresponding Author: Juebo Yu, MD, Department of Otolaryngology, Affiliated Hospital of Yangzhou University, No. 45 Taizhou Rd, Guangling District, Yangzhou 225001, China (yujuebo2004@163.com).
